# rPinecone: Define sub-lineages of a clonal expansion via a phylogenetic tree

**DOI:** 10.1099/mgen.0.000264

**Published:** 2019-03-28

**Authors:** Alexander M. Wailan, Francesc Coll, Eva Heinz, Gerry Tonkin-Hill, Jukka Corander, Nicholas A. Feasey, Nicholas R. Thomson

**Affiliations:** ^1^​ Parasites and Microbes, Wellcome Trust Sanger Institute, Wellcome Genome Campus, Hinxton CB10 1SA, UK; ^2^​ London School of Hygiene & Tropical Medicine, Keppel St., London WC1E 7HT, UK; ^3^​ Department of Mathematics and Statistics, University of Helsinki, Helsinki, Finland; ^4^​ Department of Biostatistics, University of Oslo, Oslo, Norway; ^5^​ Liverpool School of Tropical Medicine, Pembroke Pl, Liverpool L3 5QA, UK; ^6^​ Malawi Liverpool Wellcome Trust Clinical Research Programme, Blantyre, Malawi

**Keywords:** R, epidemiology, phylogenetic tree, lineage definition, methicillin-resistant *Staphylococcus aureus*, *Salmonella* Typhi

## Abstract

The ability to distinguish different circulating pathogen clones from each other is a fundamental requirement to understand the epidemiology of infectious diseases. Phylogenetic analysis of genomic data can provide a powerful platform to identify lineages within bacterial populations, and thus inform outbreak investigation and transmission dynamics. However, resolving differences between pathogens associated with low-variant (LV) populations carrying low median pairwise single nucleotide variant (SNV) distances remains a major challenge. Here we present rPinecone, an R package designed to define sub-lineages within closely related LV populations. rPinecone uses a root-to-tip directional approach to define sub-lineages within a phylogenetic tree according to SNV distance from the ancestral node. The utility of this software was demonstrated using both simulated outbreaks and real genomic data of two LV populations: a hospital outbreak of methicillin-resistant *Staphylococcus aureus* and endemic *Salmonella* Typhi from rural Cambodia. rPinecone identified the transmission branches of the hospital outbreak and geographically confined lineages in Cambodia. Sub-lineages identified by rPinecone in both analyses were phylogenetically robust. It is anticipated that rPinecone can be used to discriminate between lineages of bacteria from LV populations where other methods fail, enabling a deeper understanding of infectious disease epidemiology for public health purposes.

## Data Summary

1. Source code for rPinecone is available on GitHub under the open source licence GNU GPL 3 (url: https://github.com/alexwailan/rpinecone).

2. Newick format files for both phylogenetic trees have been deposited in Figshare (url: https://doi.org/10.6084/m9.figshare.7022558).

3. Geographical analysis of the *S*. Typhi dataset using Microreact is available at https://microreact.org/project/r1IqkrN1X.

4. Accession numbers, metadata and sample lineage results of both datasets used in this paper are listed in the supplementary tables.

5. The R code showing the simulation performed to compare rPinecone to hierBAPS as well as SNV distances, and to assess the support of the obtained clusters based on bootstrapped trees, is given as a supplementary rmarkdown file on the rPinecone GitHub site.

**Impact Statement**

Whole genome sequence data from bacterial pathogens are increasingly being used in the epidemiological investigation of infectious disease, both in outbreak and in endemic situations. However, distinguishing bacterial clones which are very similar and which are likely to be sampled within a limited spatio-temporal range presents a major technical challenge for epidemiologists. rPinecone was designed to address this challenge and utilizes phylogenetic information to distinguish extant lineages of bacterial populations. This approach is therefore of great interest to epidemiologists as it adds a further level of clarity above and beyond that offered by existing approaches which have not been designed to consider bacterial isolates containing variation that only transiently exists, but which is epidemiologically informative. rPinecone has the flexibility to be applied to multiple pathogens and has direct application for investigations of clinical outbreaks and endemic disease to understand transmission dynamics or geographical hotspots of disease.

## Introduction

Advances in whole genome sequencing (WGS) permit the study of bacteria at high resolution and this has created the opportunity to use WGS data to reliably discriminate between bacteria that were previously indistinguishable by alternative means, or to ‘type’ them. Using WGS data to understand the epidemiology of infections presents distinct challenges compared with those faced by evolutionary biologists, more so when aiming to distinguish lineages of bacterial populations that are only transiently extant.

Single nucleotide variants (SNVs) which arise stochastically may offer no evolutionary benefit to a given bacterial isolate, and therefore may only transiently be present in a population. Despite their lack of evolutionary significance, however, they may be epidemiologically informative in the context of localized outbreaks, especially amongst organisms that show low overall population diversity. Traditional genetic clustering algorithms such as structure [1] and baps [2, 3] are not suitable for sub-typing of low-variant (LV) bacterial populations over small timescales such as less than 3 years. They assume independence between loci and require tens to hundreds of SNVs that are shared between different lineages to confidently cluster populations. Novel bioinformatic approaches to identify sub-lineages within LV bacterial populations are therefore needed for epidemiological investigations, especially when technological advancements have made genomic studies of outbreaks increasingly common.

For genomic data, typing tools are used to identify ‘clusters’ or ‘partitions’ to define a group of isolates as a ‘lineage’ or sub-lineage within a population. These three terms are used synonymously in this context. However, to provide clarity we will use the term ‘sub-lineage’ to refer to a defined group of isolates within a population. In general, typing tools are non-tree based and fall under two broad approaches, distance-based methods which calculate a pairwise distance matrix often using the SNV distances between isolates, and model-based methods which rely on calculating a population genetic model using either Bayesian or maximum-likelihood (ML) based methods [1]. For distance methods samples are considered to be within the same cluster if their SNV distance is less than or equal to a specified SNV threshold. An alternative method is the widely used program hierBAPS [3] which, when given a multiple sequence alignment, attempts to identify the lineages of the sequences that maximizes the posterior probability of the hierBAPS model. The model is based on a multinomial Dirichlet distribution and assumes independence between SNV sites. The algorithm is applied first to identify an initial set of lineages, which is then iteratively repeated to generate subsequent sub-lineages within each of the initially defined lineages. While both types of approaches are suitable for populations where long-term historical evolution is present, neither is an appropriate option for short-term clonal expansions such as populations emerging from point source outbreaks or locally endemic disease. In the case of Bayesian approaches, the resulting sub-lineages are not expected to reflect the phylogenetic data because the underlying population genetic model has very limited power to separate such closely related isolates, whereas methods that use a pairwise SNV distance approach are unable to infer direction of evolution or common ancestry between sub-lineages, which is necessary for source and transmission investigations.

Here we present rPinecone, an R package which evaluates a phylogeny using a root-to-tip approach to define sub-lineages according to SNV distances from ancestral nodes. rPinecone also determines if two or more sub-lineages form part of a larger major lineage if they are related by enough ancestral nodes. To demonstrate this approach, rPinecone was compared to hierBAPS and SNV distance analysing a simulated dataset of closely related bacteria (very little variation in the alignment) such as is likely to be encountered in transmission analyses. We furthermore demonstrate its use on a local endemic dataset of *Salmonella enterica* subsp. *enterica* serovar Typhi (*S*. Typhi) from the H58 haplotype [4] as well as a hospital outbreak of methicillin-resistant *Staphylococcus aureus* (MRSA) ST2371 [5].

## Methods

### Data simulation

To compare rPinecone with hierBAPS and single-linkage hierarchical SNV clustering, we simulated data using outbreakr [6] with a sequence length of 1e+06, sample size 20, mutation rate 0.1 (representing a low mutation rate), and migration rate of 0.01 for five populations; for a more detailed script please see the supplementary rmarkdown file located in the package GitHub repository.

### Assigning support based on bootstrap

To incorporate uncertainty in a phylogeny we allow for rPinecone to be run separately on bootstrap replicates or Markov chain Monte Carlo (MCMC) samples from a Bayesian approach such as beast [7]. These are then combined into a co-occurrence matrix where we count the number of times two isolates appear in the same cluster across the replicates [8]. From this matrix we can then generate clusters across the replicates where rPinecone clusters the isolates together a given percentage of the time. An example of the use of this additional functionality is given in the supplementary rmarkdown file.

### Phylogenetic tree reconstruction of two case studies

Data from two studies were re-analysed to reconstruct phylogenetic trees for input into rPinecone. Mapping of reads was performed using smalt v0.7.4 (http://sanger.ac.uk/science/tools/smalt-0/) and SNVs were identified by using samtools mpileup [9]. Chromosomal phage regions were identified using the phaster (PHAge Search Tool Enhanced Release) database [10, 11]. Chromosomal recombination regions were identified using Gubbins [12]. SNVs found within these regions of the chromosome were excluded from the SNV alignment. The SNP-sites program was used to obtain an SNV alignment composed of only variable sites [13]. ML trees were reconstructed from the SNV alignment using RAxML [14] (version 8.2.8) with a GTR model of evolution. The ancestral reconstruction tool pyjar [15] (available at: http://github.com/simonrharris/pyjar) [15] was used to reconstruct the ML trees into an ML joint ancestral reconstruction (JAR) tree. Each JAR tree was used as an input for rPinecone to define the sub-lineages within respective populations. The R package for hierBAPS (rhierBAPS, available at https://github.com/gtonkinhill/rhierbaps) was used as a comparative benchmark for rPinecone when analysing each dataset, due to its frequent use in bacterial phylogenetics for lineage identification. Input data for this package were the SNV alignment used to generate the phylogenetic ML tree. A maximum depth level of five and maximum cluster number of 30 were used for the analysis.

### MRSA hospital outbreak

The original study had whole genome-sequenced the MRSA samples by Illumina MiSeq (Illumina) generating 150 bp paired-end reads [5]. These samples involved in the MRSA outbreak were of sequence type 2371, a single locus variant of ST22 [5]. In the re-analysis, the short reads of the 45 isolates identified as ST2371 were mapped against the chromosome of reference *Staphylococcus aureus* isolate HO 5096 0412, which was ST22. Reference isolate HO 5096 0412 was used as an outgroup, to root the tree, and was also removed from the phylogenetic tree. The tree was rooted on sample from patient 5 (accession no. ERR070046). Isolate metadata and lineages defined by rPinecone and rhierBAPS can be found in Table S1 (available in the online version of this article).

### Endemic *S.* Typhi in Cambodia

The original study whole genome-sequenced their *S.* Typhi isolates with an Illumina HiSeq2000 device, generating 100 bp paired-end reads as described in the original investigation [4]. For the re-analysis, the *S.* Typhi H58 isolates and the outgroup were genotyped using the Genotyphi scheme [16]. The short reads of the 203 *S*. Typhi H58 Genotyphi 4.3.1 were mapped to *S.* Typhi reference Genotyphi 4.3.1 isolate 2010 7898 (accession no. GCA_001360555). *S.* Typhi isolate Mal1017142 Genotyphi 4.1.1 was used as an outgroup to root the tree. The SNV alignment was processed to remove private SNVs before performing the Bayesian clustering analysis with hierBAPS [3, 17] using five nested levels. Microreact [18] was used to visualize spatial data with phylogenetic and rPinecone data. Isolate metadata and lineages defined by rPinecone and rhierBAPS can be found in Table S2.

## Results

rPinecone is a package developed in R and available under the open source licence GNU GPL3 at https://github.com/alexwailan/rpinecone. Input for rPinecone requires two user-specified integer thresholds for SNV distance and relatability (discussed below), and a rooted phylogenetic tree (Newick format) where the branch lengths are integers corresponding to SNV distance. One type of tree that fits this criterion is an ML JAR tree.

**Fig. 1.**
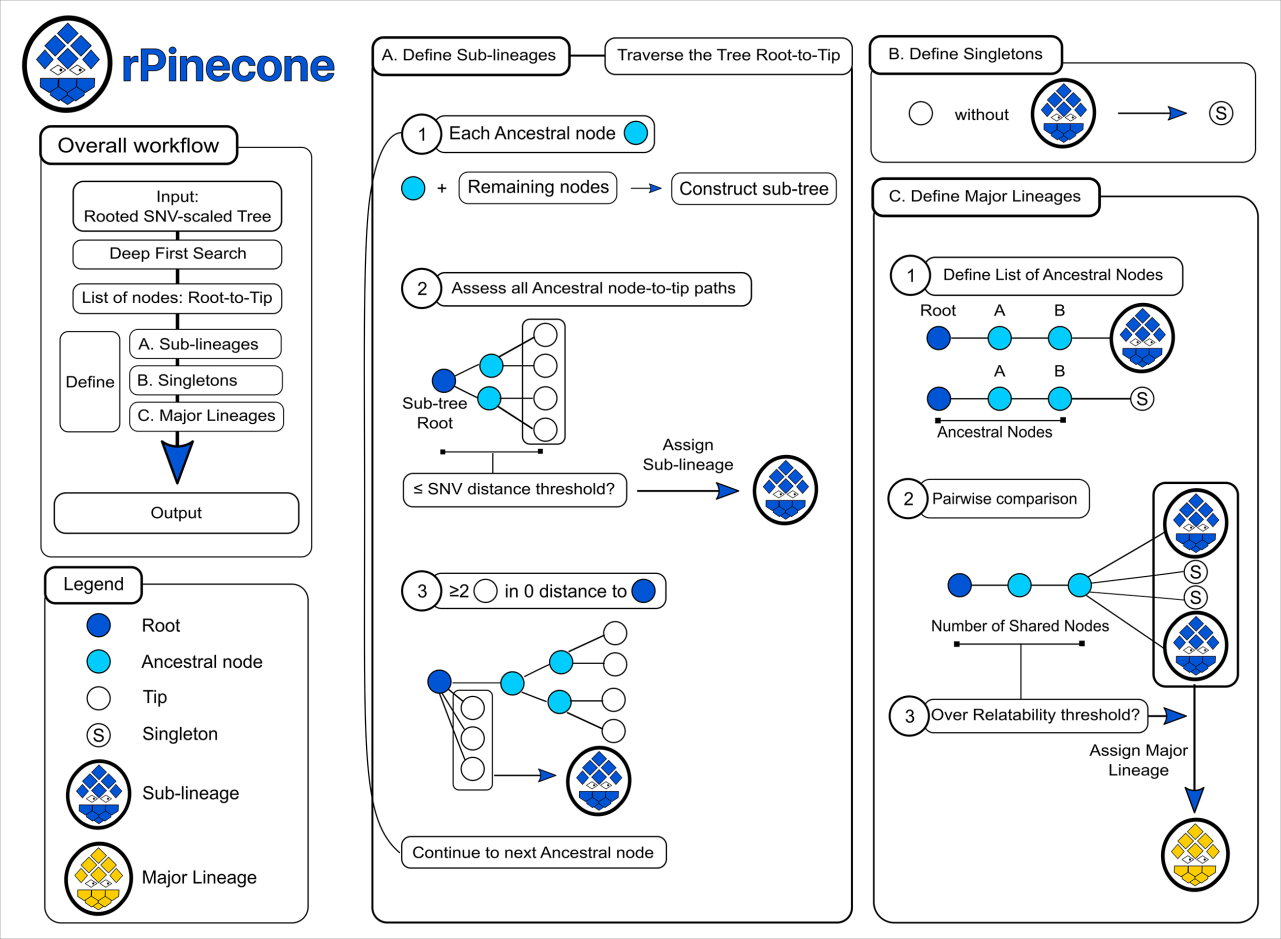
The rPinecone package evaluates a phylogenetic tree in a root-to-tip approach and defines sub-lineages according to SNV distances from ancestral nodes. (a) Define sub-lineages. The rPinecone algorithm traverses the tree from root-to-tip in the order of nodes listed by the depth-first search. At each ancestral node, a sub-tree is constructed containing the ancestral and remaining nodes. The ancestral node becomes the root of the sub-tree and all root-to-tip paths are assessed to identify the maximum root-to-tip branch distance. If this distance is below or equal to the user-specified SNV distance threshold, the tips of the sub-tree are assigned to a sub-lineage number. If the distance exceeds the threshold and two or more tips are at zero distance to the root of the sub-tree, only the tips at zero distance will be assigned a sub-lineage number. The process is repeated throughout the entire tree. (b) Define singletons. Tips which have not been assigned a sub-lineage number are assigned a singleton number. (c) Define major lineages. Using the entire tree, the list of ancestral nodes from the root to each sub-lineage and singleton is compiled. A pairwise comparison of these lists is performed. Sub-lineages and singletons which have the same number of ancestral nodes over the user-specified relatability threshold will be defined as part of a major lineage. The root, ancestral nodes and tips of the tree are denoted by navy, cyan and white circles respectively. Singletons, sub-lineages and major lineages are represented by a ‘S’ labelled circle, a blue pinecone and a gold pinecone, respectively.

The rPinecone package has a primary wrapper function to perform the analysis, which is done in two steps: sub-lineage definition and major-lineage definition (Fig. 1). rPinecone will initially prepare for sub-lineage definition by collapsing zero SNV dichotomies of the tree into polychotomies using the *ape* package [19] for R (v4.1) and performing a depth-first search (DFS) [20] across the resulting tree, to list the tips and ancestral nodes from root-to-tip. Edges are then drawn between nodes where the edge distance is the branch length, i.e. the number of SNV isolates have from an ancestral node on a JAR tree. Using the DPS and SNV branch distance information, rPinecone will then define sub-lineages by traversing the tree in a root-to-tip direction to assess each ancestral node. At each ancestral node, a sub-tree is built with the remaining nodes where the ancestral node becomes the root of the sub-tree. rPinecone uses two methods to define sub-lineages: (1) all root-to-tip paths of the sub-tree are assessed and if the maximum root-to-tip branch distance is equal to or below the specified SNV threshold, the remaining tips will be assigned a sub-lineage number; and (2) if the maximum distance is over the SNV threshold and there are at least two tips with zero distance from the ancestral node, rPinecone assigns these tips in zero distance a sub-lineage number. The process is repeated throughout the entire tree. Tips that are not assigned a sub-lineage number will be assigned with a singleton number.

After sub-lineages have been declared, their relatability is analysed to determine if they form a larger major lineage. The user-specified relatability threshold is an integer used to compare the number of ancestral nodes from the root of the entire tree to each sub-lineage and singleton. Lists of ancestral nodes for each sub-lineage and singleton are compiled. These lists are compared in a pairwise fashion. Sub-lineages and singletons that have the same intersecting ancestral nodes over the relatability threshold are declared to have formed a major lineage within the population, where a major lineage is composed of at least two sub-lineages.

Once sub-lineages and major lineages are defined, rPinecone outputs a list of six variables each of which is able to be used for downstream processes, including: the tree used for analysis, number of sub-lineages, major lineages, singletons identified and number of isolates within the tree. The final variable is a three-column table listing each ‘Taxon’ label of the tree and its respective sub-lineage and major lineage. Furthermore, phylogenetic trees can be displayed using the online display tool iTOL [21]. The rPinecone package includes three functions taking the output from the primary rPinecone function. These functions parse the rPinecone output to a file in a format to display in iTOL. Three files can be generated, a ‘LABELS’ file to change the tip labels according to their sub-lineage or singleton number as well as two ‘DATASET_COLORSTRIP’ format files to display the sub-lineages and major lineages as a colour strip/block. To show the utility of rPinecone we performed a comparison with simulated outbreak data, as well as two case studies on an MRSA hospital outbreak and local endemic dataset of *S.* Typhi.

## Simulated data to assess performance and uncertainty

**Fig. 2.**
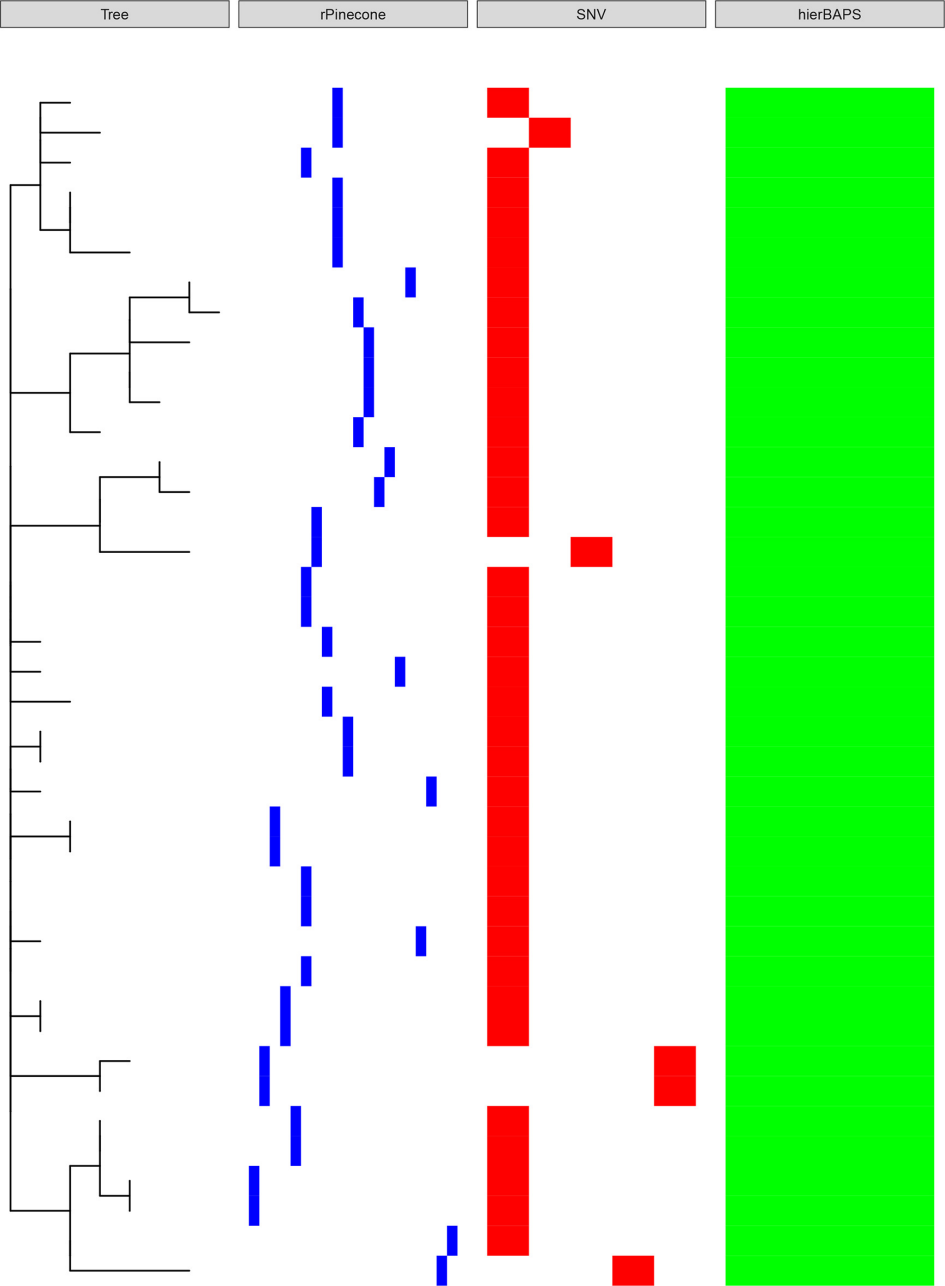
The resolution for rPinecone to identify lineages was compared with hierBAPS and single-linkage hierarchical SNV clustering. The phylogenetic tree was generated from simulated data. Panels are, from left to right: SNV-scaled phylogenetic tree, rPinecone clustering (blue), SNV single-linkage hierarchical clustering, and clustering by hierBAPS.

**Fig. 3.**
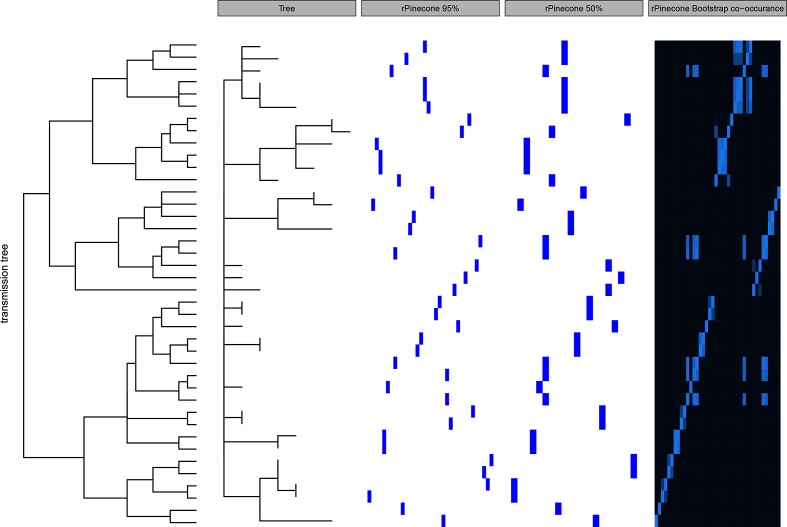
Robustness and the uncertainty of the clusters generated by rPinecone was assessed using bootstrap replicates of isolates from simulated data. Clusters were generated from a co-occurrence matrix to represent when rPinecone clusters isolates together at 50 and 95 % of the time (intervals). Panels are, from left to right: original transmission tree, SNV-scaled phylogenetic tree, rPinecone clustering at the 95 % interval, rPinecone clustering at the 50 % interval and co-occurrence matrix.

Data were simulated using outbreakr [6] to compare the performance of rPinecone with hierBAPS and single-linkage hierarchical SNV clustering (Fig. 2). This clearly shows the strength of rPinecone: being able to distinguish short, recently emerged but distinct clusters, which can be crucial in an outbreak scenario, including the distinction of branches from an unresolved node (polytomy). Both single-linkage hierarchical SNV clustering as well as hierBAPS lack the resolution necessary to disentangle closely related isolates. We furthermore used this dataset to demonstrate the function of assessing uncertainty. Given that tree calculations are always an approximation of the correct tree given the available data and choice of model, we added the possibility for the user to assess the level of uncertainty for the clusters generated, based on the bootstrap support for the given branches in the tree topology (Fig. 3).

## Hospital outbreak of MRSA ST2371 – healthcare workers were colonized by a major lineage

**Fig. 4.**
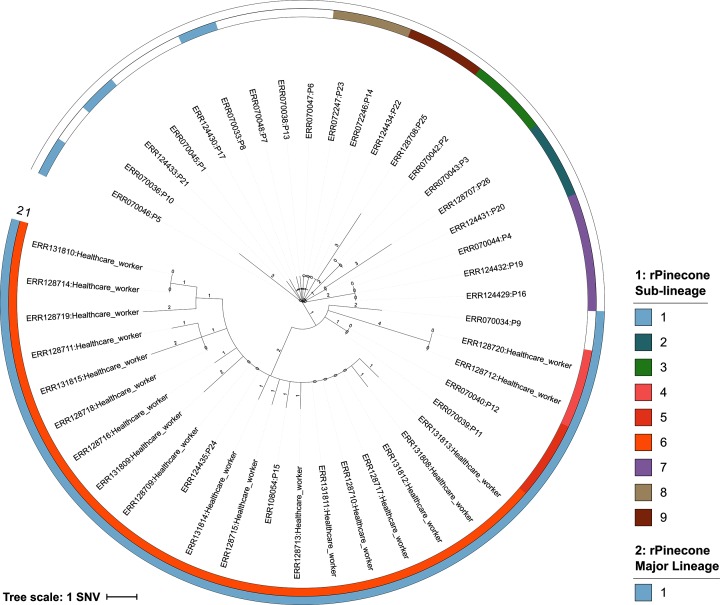
Rooted accelerated transformation tree based on WGS of 45 MRSA ST2371 isolates. The outgroup was rooted using *S. aureus* ST22 isolate HO 5096. Tips are labelled by the sample’s ERR number followed by the origin of sample, patient (P) or healthcare worker. Branch lengths are SNV-scaled. The inner and outer coloured rings correspond to the sub-lineages and major lineage identified by rPinecone, respectively.

In 2011 over a 6-month period, the National Health Service Foundation Trust in Cambridge, UK, investigated transmission of MRSA within the hospital neonatal unit and community and described a complex transmission network [5]. We re-analysed the data of this study and generated a phylogenetic ML tree. From this analysis the population had a median pairwise distance of five SNVs. The phylogenetic tree (Fig. 4) of the outbreak had a structure that begins with a root isolate (Patient 5), then expands outwards in a ‘star-burst’-like fashion where each branch of the tree represents a transmission pathway of MRSA between infants and their mothers, other mothers on the ward, and to partners of affected mothers as described in the original study [5]. rPinecone identified nine sub-lineages and a major lineage composed of three sub-lineages (25 isolates) using an SNV threshold of 4 and a relatability threshold of 3. These sub-lineages corresponded to the transmission pathways determined in the original investigation using epidemiological data. The major lineage represents the sub-lineages of MRSA (rPinecone sub-lineages 4, 5 and 6) carried by colonized healthcare workers which were associated with subsequent episodes of MRSA 64 days after the last MRSA-positive patient left the unit.

The major lineages identified by rPinecone highlight sub-lineages related by ancestral nodes and potentially lineages that have separated from the remaining clonal population. This type of analysis was not performed during the original analysis, but we successfully identified MRSA transmission pathways using rPinecone and provided an additional layer of sub-lineage data to complement the epidemiological data when associating sub-lineages with likely transmission events.

## Locally endemic *S.* typhi in rural Cambodia – higher resolution to identify circulating sub-lineages

**Fig. 5.**
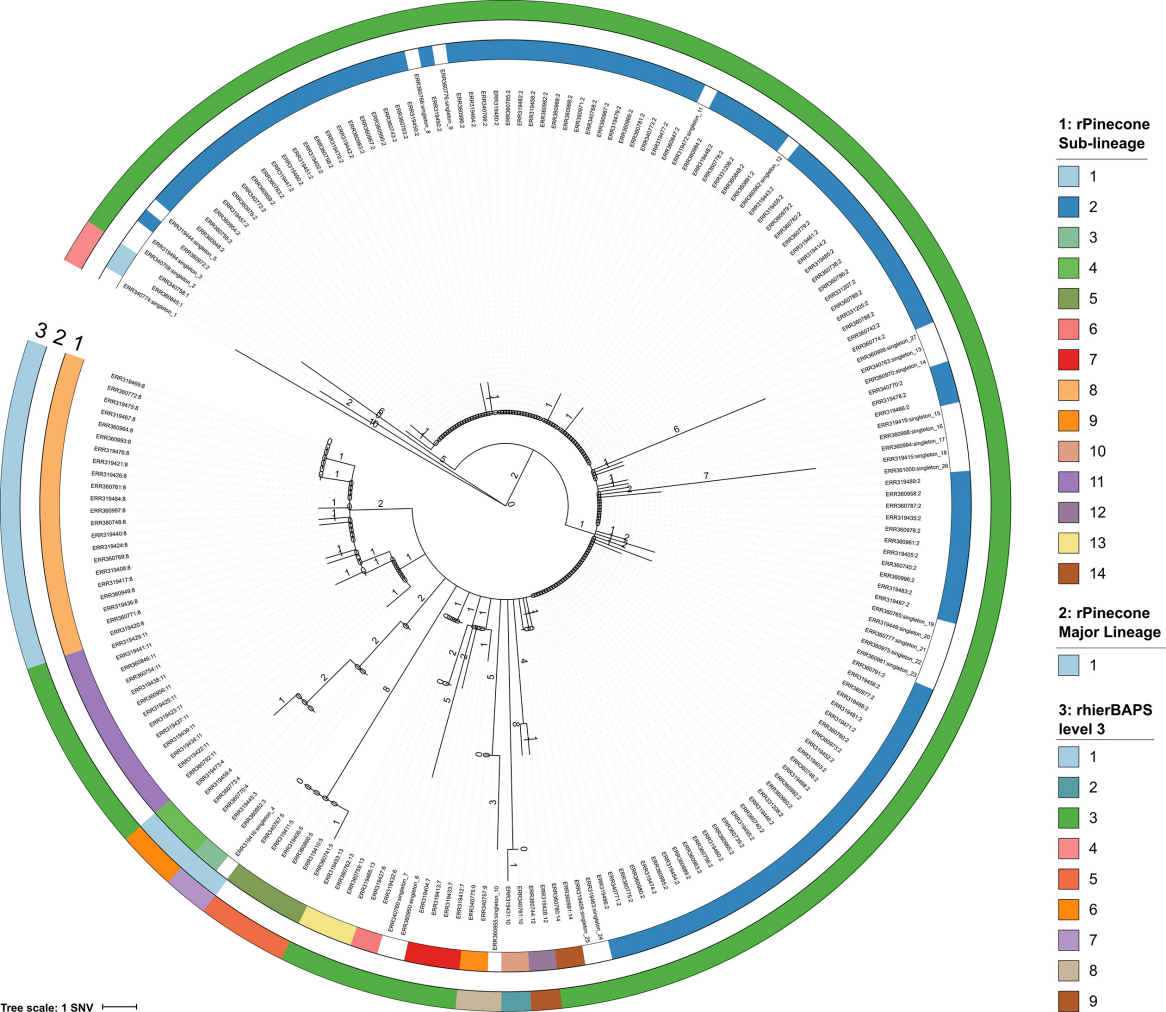
Rooted JAR tree based on WGSg of 203 *S*. Typhi H58 isolates. Outgroup was rooted using *S.* Typhi Genotyphi 4.1.1 isolate Mal1017142. Tips are labelled by the sample’s ERR number and rPinecone sub-lineage. Branch lengths are SNV-scaled. Colour rings 1 and 2 correspond to the sub-lineages and major lineage defined by rPinecone, respectively. Colour ring 3 corresponds to clusters defined by rhierBAPs level 3.

In the second example, rPinecone distinguished lineages of an *S*. Typhi population circulating in a defined geographical area causing locally endemic disease, as opposed to a clonal outbreak. LV bacterial pathogens responsible for localized, endemic disease present a different challenge; in this context, it is highly likely that multiple, genetically similar sub-lineages will be in circulation, and in order to be epidemiologically informative, phylogenetic analysis must be able to distinguish these lineages despite the low variation between them. Whilst hierBAPS is unable to utilize the LV SNV information to define lineages, rPinecone can be used in such situations. We demonstrate this by defining sub-lineages in an LV population of *S.* Typhi H58 responsible for endemic typhoid fever in a rural part of Cambodia [4]. Re-analysis of these data determined the population to have a median pairwise SNV distance of 2. The subsequent phylogenetic tree of the re-analysis can best be described from root-to-tip (Fig. 5). At the root of the tree is a ‘root group’ of isolates, then a main branch consisting of identical isolates which can be referred to as the ‘primary group’ of the clonal expansion, followed by ‘diverging groups’ of isolates and singletons which have diverged from this main branch. Using the re-analysed data, rPinecone identified 14 sub-lineages and a major lineage composed of two sub-lineages (seven isolates) of *S.* Typhi, using an SNV threshold of 2 and a relatability threshold of 3 (Fig. 5). In total, 27 isolates were also identified as singletons. By contrast, rhierBAPS identified a maximum of nine sub-lineages at level 3 and offered no further resolution after further analysis (2, 7, 9, 9 and 9 sub-lineages were identified at levels 1–5, Fig. S1). rPinecone sub-lineages were nested within the sub-lineages defined by rhierBAPS. rPinecone also acknowledges singleton isolates which have accumulated their own ‘private’ SNVs. Therefore, singletons that have individual SNVs may be excluded in the definition of sub-lineages. In contrast, rhierBAPS cannot detect the private SNVs of singletons. This is because rhierBAPs seeks to maximize the posterior probability of a sub-lineage assuming independence between SNV sites, and is less suitable for the identification of singletons. In addition, rhierBAPS also identifies isolates situated across the tree to be members of the same sub-lineage. hierBAPS and other similar programs such as structure are not designed to separate population data that would be observed towards the tips of a phylogenetic tree including those observed with a median pairwise SNV of 2 as observed here. Rather, they aim to cluster isolates into groups that are likely to come from similar source populations. When there are few isolates and few SNVs there is little information to estimate the likely distribution of allele frequencies in these source populations.

In this example, rPinecone provided greater resolution to distinguish sub-lineages than across five different levels of rhierBAPS and distinguished additional sub-lineages. The original study noted their sub-lineages to have significant geographical variation. The rPinecone sub-lineages provided a cross-sectional view of the bacterial population, where some sub-lineages were geographically confined to districts within one province and others span multiple provinces (Fig. S2).

## Conclusion

Investigators must be able to distinguish isolates to identify epidemiologically informative sub-lineages, but this is difficult for bacterial populations associated with both outbreaks and endemic disease over small temporal or geographical distances when the pathogen has a slow mutation rate and appears to be largely ‘clonal’. The ability to make this distinction is critical if WGS data are to inform epidemiological investigation of such pathogens, whether in epidemic or locally endemic disease. When aiming to locate hotspots of disease transmission geographically, it is critical to be able to incorporate geospatial data associated with samples. rPinecone was particularly designed for the analysis of LV populations and to be applied to any bacterial species with at least a median SNV distance of 2 within the population. We demonstrate the increase in resolution rPinecone provides compared to two widely used methods, hierBAPS and single-linkage hierarchical SNV clustering, in a simulated dataset of an outbreak with closely related lineages. Using the genomic data of an MRSA hospital outbreak and endemic *S.* Typhi, we furthermore demonstrate that rPinecone can be used to identify sub-lineages within LV populations that are reflective of the phylogenetic data. Furthermore, when compared to rhierBAPS, rPinecone identified additional sub-lineages. We highly recommend using hierBAPS for initial analysis of phylogenetic data to understand the bacterial population. Once this has been done, rPinecone will define sub-lineages of LV bacterial populations to set the platform for understanding the transmission dynamics or geographical hotspots of disease.

## Data Bibliography

1. Holden, M. T. *et al*., *Staphylococcus aureus* subsp. *aureus* str. HO 5096 0412, 2015, accession number HE681097.
2. Pham Thanh, D, *et al*., *Salmonella enterica* subsp. *enterica* serovar Typhi str. Mal1017142, 2016, accession number ERR279139.
3. Pham Thanh, D, *et al*., *Salmonella enterica* subsp. *enterica* serovar Typhi str. 2010_7898, 2016, accession number GCA_001360555.

## Supplementary Data

Supplementary File 1

Supplementary File 2

Supplementary File 3

## References

[1] Pritchard JK, Stephens M, Donnelly P (2000). Inference of population structure using multilocus genotype data. Genetics.

[2] Corander J, Waldmann P, Sillanpää MJ (2003). Bayesian analysis of genetic differentiation between populations. Genetics.

[3] Cheng L, Connor TR, Sirén J, Aanensen DM, Corander J (2013). Hierarchical and spatially explicit clustering of DNA sequences with BAPS software. Mol Biol Evol.

[4] Pham Thanh D, Thompson CN, Rabaa MA, Sona S, Sopheary S (2016). The molecular and spatial epidemiology of typhoid fever in rural Cambodia. PLoS Negl Trop Dis.

[5] Harris SR, Cartwright EJ, Török ME, Holden MT, Brown NM (2013). Whole-genome sequencing for analysis of an outbreak of meticillin-resistant *Staphylococcus aureus*: a descriptive study. Lancet Infect Dis.

[6] Jombart T, Cori A, Didelot X, Cauchemez S, Fraser C (2014). Bayesian reconstruction of disease outbreaks by combining epidemiologic and genomic data. PLoS Comput Biol.

[7] Bouckaert R, Heled J, Kühnert D, Vaughan T, Wu CH (2014). BEAST 2: a software platform for Bayesian evolutionary analysis. PLoS Comput Biol.

[8] Strehl A, Ghosh J (2003). Cluster ensembles - a knowledge reuse framework for combining multiple partitions. J Mach Learn Res.

[9] Li H, Handsaker B, Wysoker A, Fennell T, Ruan J (2009). The sequence alignment/map format and SAMtools. Bioinformatics.

[10] Arndt D, Grant JR, Marcu A, Sajed T, Pon A (2016). PHASTER: a better, faster version of the PHAST phage search tool. Nucleic Acids Res.

[11] Zhou Y, Liang Y, Lynch KH, Dennis JJ, Wishart DS (2011). PHAST: a fast phage search tool. Nucleic Acids Res.

[12] Croucher NJ, Page AJ, Connor TR, Delaney AJ, Keane JA (2015). Rapid phylogenetic analysis of large samples of recombinant bacterial whole genome sequences using Gubbins. Nucleic Acids Res.

[13] Page AJ, Taylor B, Delaney AJ, Soares J, Seemann T (2016). *SNP-sites*: rapid efficient extraction of SNPs from multi-FASTA alignments. Microb Genom.

[14] Stamatakis A (2014). RAxML version 8: a tool for phylogenetic analysis and post-analysis of large phylogenies. Bioinformatics.

[15] Pupko T, Pe'er I, Shamir R, Graur D (2000). A fast algorithm for joint reconstruction of ancestral amino acid sequences. Mol Biol Evol.

[16] Wong VK, Baker S, Connor TR, Pickard D, Page AJ (2016). An extended genotyping framework for *Salmonella enterica* serovar Typhi, the cause of human typhoid. Nat Commun.

[17] Corander J, Waldmann P, Marttinen P, Sillanpää MJ (2004). BAPS 2: enhanced possibilities for the analysis of genetic population structure. Bioinformatics.

[18] Argimón S, Abudahab K, Goater RJ, Fedosejev A, Bhai J (2016). Microreact: visualizing and sharing data for genomic epidemiology and phylogeography. Microb Genom.

[19] Paradis E, Claude J, Strimmer K (2004). APE: analyses of phylogenetics and evolution in R language. Bioinformatics.

[20] Prosperi MC, Ciccozzi M, Fanti I, Saladini F, Pecorari M (2011). A novel methodology for large-scale phylogeny partition. Nat Commun.

[21] Letunic I, Bork P (2016). Interactive tree of life (iTOL) v3: an online tool for the display and annotation of phylogenetic and other trees. Nucleic Acids Res.

