# Supplementary material for: rPinecone: Define sub-lineages of a clonal expansion via a phylogenetic tree
Source: Microb Genom. 2019 Mar 28;5(4):e000264. doi: 10.1099/mgen.0.000264 (PMC6521585; doi:10.1099/mgen.0.000264)
Supplement: Supplementary File 1 [file mgen-5-264-s001.pdf]

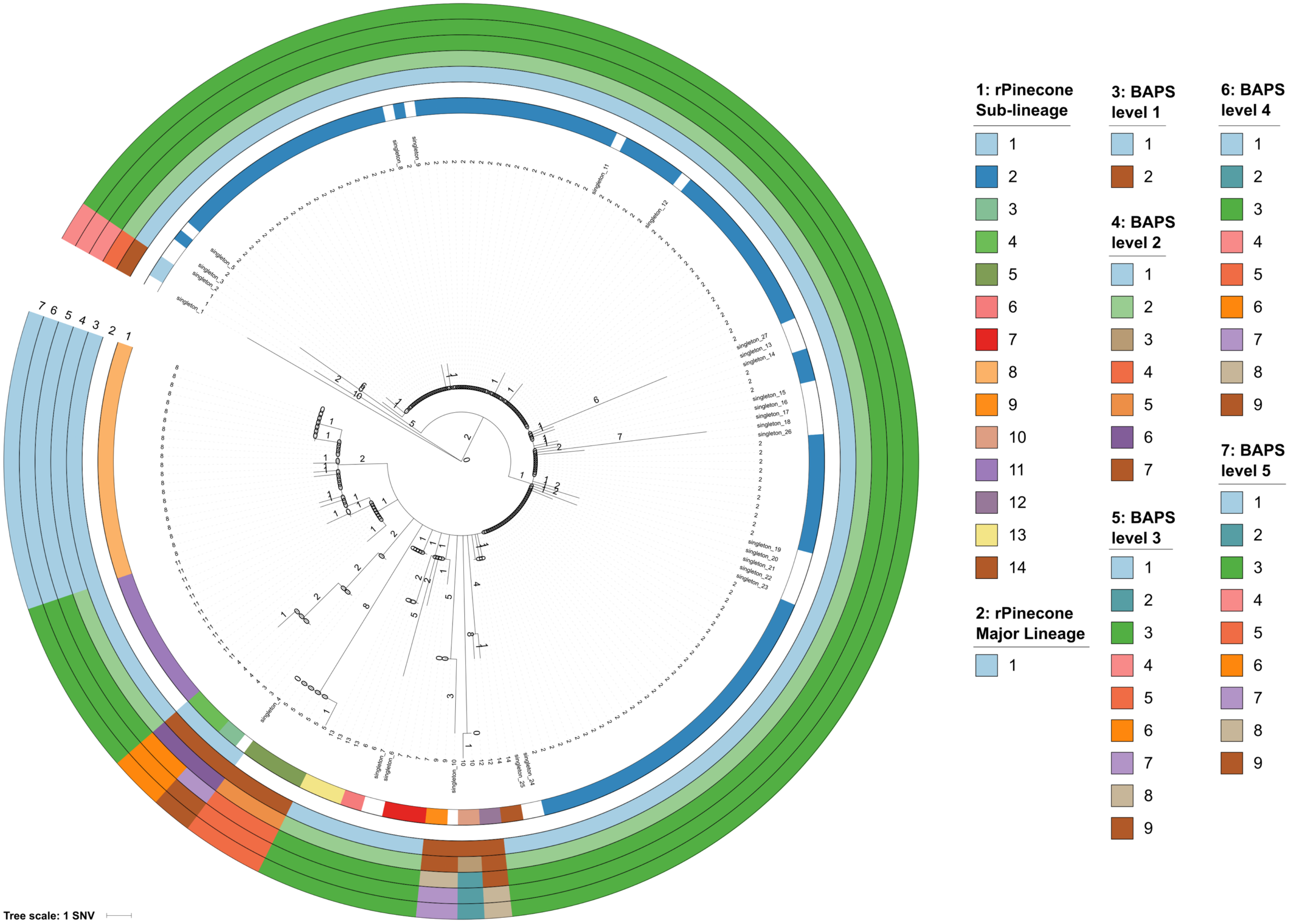
1: rPinecone Sub-lineage
1
2
3
4
5
6
7
8
9
10
11
12
13
14
2: rPinecone Major Lineage
1
3: BAPS level 1
1
2
4: BAPS level 2
1
2
3
4
5
6
7
5: BAPS level 3
1
2
3
4
5
6
7
8
9
6: BAPS level 4
1
2
3
4
5
6
7
8
9
7: BAPS level 5
1
2
3
4
5
6
7
8
9
Tree scale: 1 SNV

Supplementary Figure 1. Rooted joint ancestral reconstruction tree based on whole genome sequencing of 203 *S*. Typhi H58 isolates. Outgroup rooted using *S.* Typhi Mal1017142 Genotyphi 4.1.1. Tips are labelled according to rPinecone sub-lineage. Branch lengths are SNV scaled. Colour rings 1 and 2 correspond to the sub-lineages and major lineage defined by rPinecone. The five outer (3-7) colour rings correspond to clusters defined by BAPs.

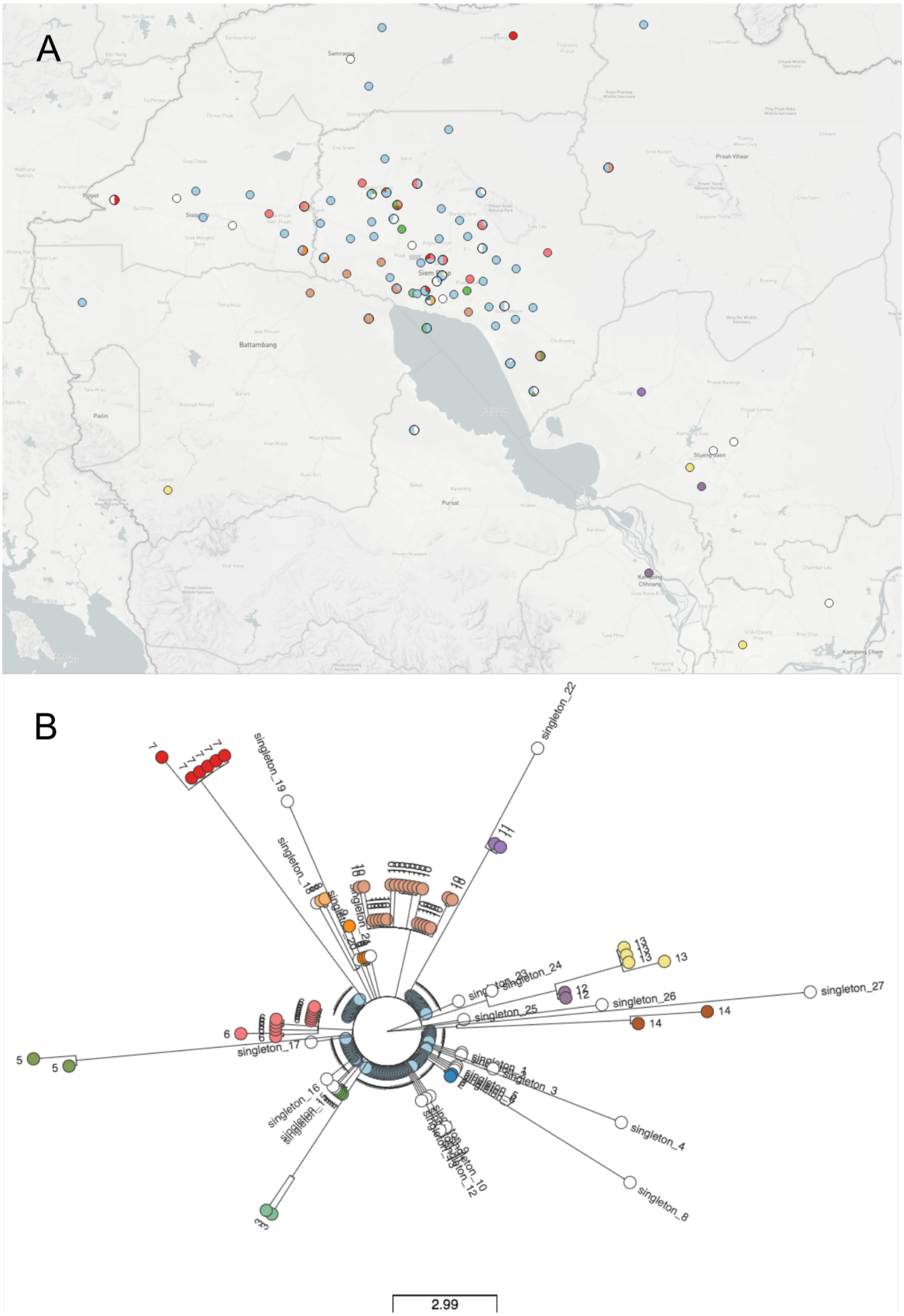
A
Samraong
Battambang
Pursat
Preah Vihear
Kampong Cham
B
singleton_22
singleton_19
singleton_27
singleton_26
singleton_25
singleton_17
singleton_16
singleton_4
singleton_8
2.99

Supplementary Figure 2. Isolate spatial, phylogenetic and clustering data displayed in Microreact. A) Geospatial distribution of *Salmonella* Typhi isolates in Cambodia. B) The phylogenetic tree with isolates labelled with their corresponding sub-lineage defined by rPinecone. Isolates in both panels are coloured according to sub-lineages.
